# Detector Processor for a 5G Base Station

**DOI:** 10.3390/s22207731

**Published:** 2022-10-12

**Authors:** Cao Niu, Dake Liu

**Affiliations:** State Key Laboratory of Marine Resource Utilization in South China Sea, School of Information and Communication Engineering, Hainan University, Haikou 570228, China

**Keywords:** MIMO, OFDM, K-best, signal detection

## Abstract

Nonlinear soft bit detection is essential for the uplink receivers of 5G base stations, especially for users around the cell edge. However, its throughput and computing complexity are always challenges for both research and industry. A low-cost and low-power parallel implementation of a soft-output detector based on sorted QR decomposition (SQRD) and the K-best breadth-first search algorithm is thus proposed to reduce computational complexity and latency. In addition, to save area and reduce latency, two improvement methods are used: (1) reduce the computing cost by saturating and truncating large values during PED computing and (2) reduce the sorting cost by using the binary bit sorting method for a reduced sample set with finite accuracy. Furthermore, a pipelined VLSI architecture is designed using 28-nm digital CMOS technology offered by Semiconductor Manufacturing International Corporation (SMIC). It can achieve a peak throughput of 6400 Mbps while consuming 153 K gates (including all flip-flops) for SMIC’s 28-nm technology and running at 800 MHz, a 32% cost reduction compared with the published reference design.

## 1. Introduction

5G technology requires great bandwidth, a fast data transmission rate and very reliable transmission performance. The detector processor implemented in the past can no longer meet the needs of signal detection in 5G micro base stations. How to reduce the hardware overhead and scale while ensuring the demodulation performance so that it can be well applied in 5G micro base stations is an important problem. The performance of nonlinear detection is better than that of linear detection in multiple-input multiple-output (MIMO) systems [1]. Its computational complexity is high at the same time. A low-cost, high-throughput and near-optimal MIMO detector has always been the key to the designing a receiver [2]. The maximum likelihood (ML) algorithm can theoretically provide the best detection performance. In [3,4,5], the exhaustive search method was suitable for a small modulation scale, and the computational complexity was unacceptable when the modulation scale was large. In order to reduce the computational complexity, a nonlinear detection method, spherical decoding(SD) based on tree searching, is widely used. For example, the width-first (K-best) method was used in [6,7,8,9,10], the depth-first method was used in [11,12], and so on. By properly controlling the number of nodes at each search level, it has near-optimal performance and scalable complexity. The K-best algorithm is a forward algorithm with a fixed throughput, so it is suitable for the implementation of a pipelined architecture. However, when the size of the antenna and signal modulation are large, in order to be close to the ML algorithm, the K value often needs to be very large, so the nodes that need to be calculated in the search will be doubled, thus increasing the amount of computation. Therefore, in the design of 5G micro base station receivers, the selection of candidate nodes needs a new method to further improve.

A few implementations using field-programmable gate arrays (FPGAs) and application-specific integrated circuits (ASICs) have been designed based on K-best detectors. For example, the authors of [13] used the K-best method of an adaptive radius for hardware implementation, but this requires constant backtracking to calculate the radius and control the number of candidate nodes, which is not efficient enough. In [14], although the K-best algorithm could achieve a high throughput, it was not flexible enough, and the hardware cost was too high. In [15,16], preprocessing and parallel computing were used to improve the SD algorithm, but the complexity of the SD algorithm itself would lead to a waste of hardware resources and higher computing time cost. In order to further improve the bit error rate performance, a soft output detector [17] which can generate a logarithmic likelihood ratio (LLR) is proposed. It can be seen that the demodulation performance was improved, but it brought an unnecessary hardware overhead and needs further improvement. However, in most of the references, the hardware and computing costs of the aforementioned solutions were high and hard to accept in 5G base stations. In this paper, we focus on cost reduction while maintaining near-optimum performance.

In this paper, the soft-output K-best MIMO detector based on sorted QR decomposition (SQRD) was improved and implemented, with low complexity and near-optimum performance as well support for different kinds of modulation. Improvements include data saturation for partial Euclidian distance (PED) calculation and a bit sorting method for small-sample binary data with finite accuracy. Our design is verified by its synthesized RTL design and silicon layout. The IC layout is based on the COMS technology of SMIC (28 nm). The circuit achieves a peak throughput of 6400 Mbps at 800 MHz while consuming 153 K gates, with 55 cycles for latency. It is better than the currently published solutions and can meet the requirements of 5G micro base station uplink receivers.

This paper is organized as follows. Section 2 reviews the frequency domain model of the MIMO system and K-best decoding algorithm. Section 3 discusses the modified K-best decoding algorithm and presents the performance simulation results. Section 4 describes the hardware design and implementation results of the proposed algorithm. The conclusion is given in Section 5.

## 2. System Model

**Figure 1:**
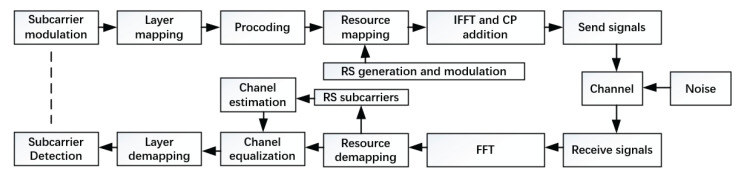
The flow chart of the 5G baseband system.

The system in this paper actually refers to the 5G micro base station system, for which a a flow chart of the 5G baseband system is shown in Figure 1.

First, the data are modulated on subcarriers, and the data stream is mapped to the constellation. At the same time, the RS reference signal is generated and modulates other subcarriers, which is convenient for the receiver to estimate the channel distortion. After layer mapping, resource mapping and precoding, IFFT and other steps, the modulated subcarriers in the F domain are converted into symbols in the time domain that can be transmitted by the digital front end. Together with the noise interference and channel distortion, the transmitted symbols are received at the front. After FFT and de-mapping processing, the transmitted symbols are converted into a subcarrier data signal for subsequent processing. The channel is estimated by analyzing the RS reference signal and noise, and the channel matrix H of each data subcarrier is equalized by interpolation or Wiener filtering. Finally, the received signal is detected and decoded.

### 2.1. Soft-Output Detection

Consider a MIMO system with M transmitting and N receiving antennas. The complex equivalent model can be expressed as [18]

(1) $$\begin{array}{cc} y & =H\cdot s+n \end{array}$$

where s is the M dimensional transmit symbol vector and each element is modulated by Mc coded bits. H is an N × M channel matrix, and n is independent and identically distributed complex Gaussian noise, while y is the received symbol vector.

Each symbol in s is drawn from the real constellation W. H can be decomposed by QR decomposition into a unitary matrix Q and an upper triangular matrix R [19]. Then, the objective of MIMO detection is to find the closest possible transmitted vector $\hat{s}$:

(2) $$\begin{array}{cc} \hat{s} & =\underset{s\in \Omega^{M}}{\arg\min}{∥\hat{y}-\mathrm{Rs}∥}^{2} \end{array}$$

where $\hat{y}$ is $Q^{H}y$, with $Q^{H}y$ being the conjugate transpose of Q.

The soft-output MIMO detector generates a posteriori probability for each bit by calculating the extrinsic LLR values. The LLR of bit $x_{i}$ is defined as [20,21]

(3) $$\begin{array}{cc} \mathrm{LLR}\left(x_{i}|y\right) & =\ln\frac{P\left(x_{i}=1|y\right)}{P\left(x_{i}=0|y\right)}\\ \; & \approx \frac{1}{2\sigma^{2}}\left\{\min_{X_{i}^{1}}∥\hat{y}{-\mathrm{Rs}∥}^{2}-\min_{X_{i}^{0}}{∥\hat{y}-\mathrm{Rs}∥}^{2}\right\} \end{array}$$

### 2.2. K-Best Algorithm

K-best is an optimization algorithm based on the breadth-first tree search algorithm. For M-QAM modulation, the K-best algorithm can be described as the following operations:

1. Extension: Store the candidate child nodes of each layer in the node collection as candidate nodes of each layer;
2. Calculation: The PED is calculated based on the root node and its candidate child nodes;
3. Sorting: When the PED values of all candidate child nodes in a layer are calculated, the sorting operation is carried out, and the smallest K nodes are retained as the parents of the next layer’s candidate nodes. If this is not the last layer, then return to step 2.

According to the flow of the K-best algorithm, we can see that the whole of path expansion is a forward process. Therefore, algorithm-level parallelism can be realized. When the K-best detector needs to calculate the soft output, the reserved paths can be easily used to generate an ordered candidate list.

## 3. Algorithm Implementations

### 3.1. Algorithm Description

In this paper, the soft-output K-best MIMO detector based on SQRD is proposed which combines the advantages of the K-best algorithm and spherical decoding algorithm and can achieve a performance close to the ML algorithm, even after significantly reducing the number of search nodes.

In K-best detection, the channel matrix H should be decomposed by QR first. The i-th column of the channel matrix H represents the transmission function from the i-th transmitting antenna to multiple receiving antennas, so the column norm of the channel matrix is the strength of the transmitted signal [22,23]. Before the QR decomposition of the channel matrix H, the column norm of H is calculated, and the channels are sorted from weak to strong according to the column norm order from small to large. The QRD of the sorted H ensures that the order of signal detection is carried out according to the SNR from high to low, namely in SQRD detection.

When detecting the signals layer by layer, to maintain performance with a small K, and to minimize the candidate nodes computing in each layer of the K-best calculation as much as possible, it is necessary to limit the number of candidate nodes in each layer of the K-best algorithm [24]. In the algorithm designed in this paper, the nearest 16 nodes within a square, as the received signal, are selected as the expansion nodes by trading off performance and cost. At the same time, to improve the expansion node accuracy, for the received signal, the MMSE channel equalization is preprocessed to solve the initial estimate $S_{\mathrm{MMSE}}$, and the nearest 16 nodes are selected as the expansion nodes of each layer to participate in the operation. The MMSE algorithm is used to limit the region of the candidate nodes, which greatly reduces the number of nodes needed in demodulation. The volume results show that the reduction in candidate nodes does not affect the performance in the case of a low SNR. Therefore, the search space reduction in this paper gives a negligible performance penalty.

**Figure 2:**
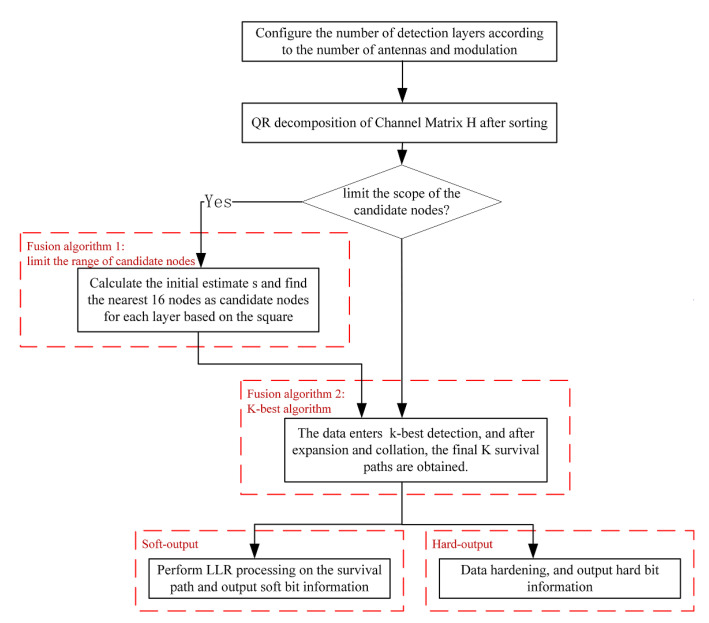
Flow chart of the proposed algorithm in this paper.

Within the range of candidate nodes limited by the SD and MMSE, using the K-best algorithm can greatly reduce the number of nodes to be calculated, reduce the computing time, and ensure the BER performance in 64-QAM and 256-QAM high-order constellation demodulation. The flow chart of the system algorithm designed in this paper is shown in Figure 2.

There are M transmitting antennas and N receiving antennas. The transmitter sends the signal $s_{M\times l}$, the receiver receives the signal $y_{N\times l}$, and the channel estimation matrix is $H_{N\times M}$. For the received signal $y_{N\times l}$ and channel estimation matrix $H_{N\times M}$, the following steps are employed:

*Step 1.* Deal with the H matrix: The column norm of the H matrix is solved and rearranged from small to large according to the norm value, and the $H^{\prime }$ and permutation matrix P are obtained.

*Step 2.* QR decomposition: The Q matrix and R matrix are obtained by QR decomposition of $H^{\prime }$.

*Step 3.* Calculate the MMSE channel equalization matrix [25] as follows:

(4) $$\begin{array}{cc} G_{MMSE(N\times M)} & ={\left(H^{H}H+\sigma^{2}I\right)}^{-1}H^{H} \end{array}$$

*Step 4.* Deal with the received signal y:

(5) $$\begin{array}{cc} Y_{\left(M\times 1\right)} & =Q_{\left(M\times N\right)}^{-1}y_{\left(N\times 1\right)} \end{array}$$

*Step 5.* Calculate the initial estimated signal:

(6) $$\begin{array}{cc} S_{MMSE(N\times 1)} & =G_{MMSE(N\times M)}Y_{\left(M\times 1\right)} \end{array}$$

*Step 6.* Extended candidate node: With $S_{MMSE(N\times 1)}$ as the center, a maximum of 16 constellation points is extended as candidate child nodes. The N layer presets up to 16 child nodes per layer.

*Step 7.* Enter the K-best algorithm: The PED calculation formula for each layer is

(7) $$\begin{array}{c} \Delta_{i}=\left|y_{i}-\sum_{j=i+1}^{N}R_{ij}S_{j}-R_{ii}S_{i}\right|+\Delta_{i+1} \end{array}$$

*Step 8.* Hard output: After step 7, the path value of each layer corresponding to the shortest PED value is directly output, which is the hard output.

*Step 9.* Soft output: Calculate the LLR value of each bit and output, where the formula is as follows:

(8) $$\begin{array}{c} \mathrm{LLR}\left(x_{i}|y\right)\approx \frac{1}{2\sigma^{2}}\left\{\min_{X_{i}^{1}}∥\hat{y}{-\mathrm{Rs}∥}^{2}-\min_{X_{i}^{0}}{∥\hat{y}-\mathrm{Rs}∥}^{2}\right\} \end{array}$$

### 3.2. Algorithm Simulation Results

A MIMO detection system suitable for a 5G micro base station was built on the Matlab platform, and the antenna size was set to four to transmit and four to receive. To generate source data on the transmitter side, the binary code stream was randomly generated as the signal, the ZC sequence was used as the reference signal, and the reference signal subcarriers were modulated as BPSK. To build a channel, the channel environment was the Rayleigh channel with steady fading, and the corresponding multipath effect parameters were set to simulate the addition of noise interference in the channel. At the receiving side, the channel was estimated based on the pilot subcarriers, and interpolation and channel equalization were performed between subcarriers at the non-pilot subcarriers in the frequency domain.

The related verification of different detection algorithms was carried out in the built system. For the verification of the improved algorithm designed in this paper, several test functions were written, which were the traditional K-best algorithm function, the QRD preprocessing function of the H matrix, the SQRD preprocessing function of the H matrix, the K-best algorithm function of a simplified search space, the hard output function, the soft LLR calculation function and so on.

**Figure 3:**
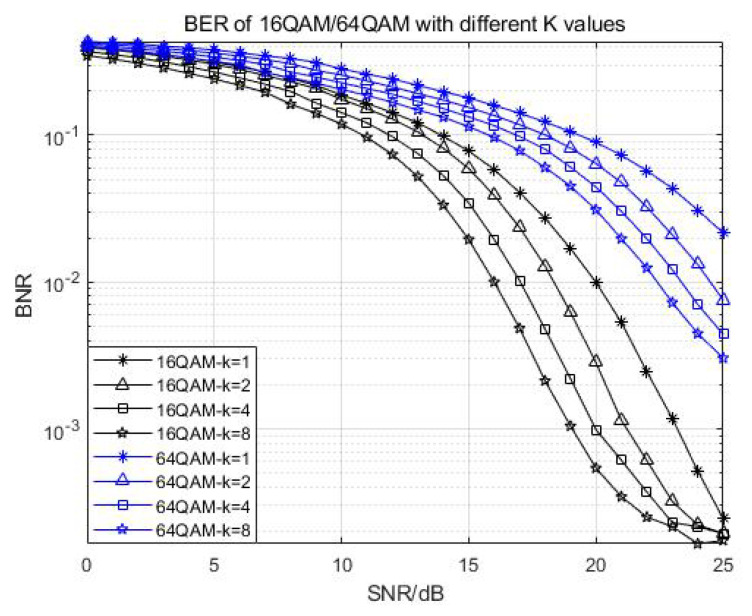
BER of 16-QAM and 64-QAM demodulation with different K values.

**Figure 4:**
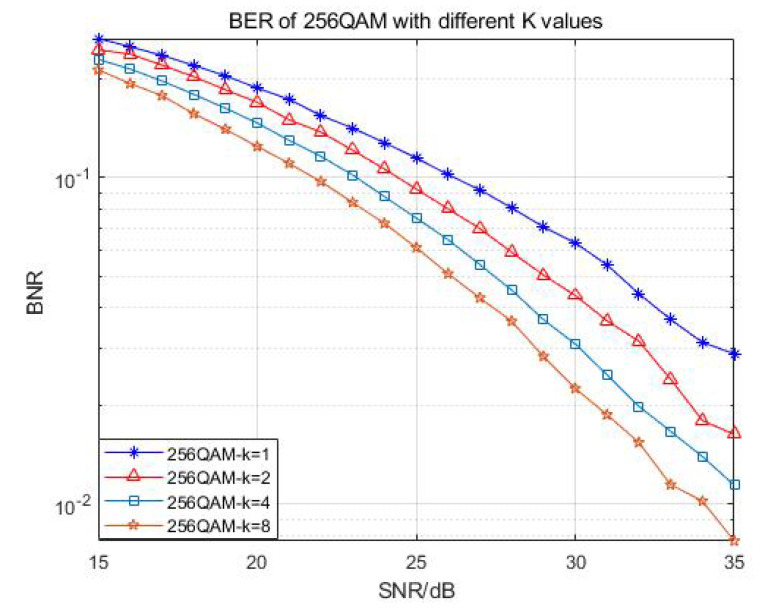
BER of 256-QAM demodulation with different K values.

The above functions were tested, and the relevant bit error rate was calculated to verify the correctness of the behavior model. Compared with the ML algorithm, the performance reduction of the algorithm we used was between 0.5 and about 1 dB, which could ensure the effectiveness of the algorithm. After finalizing the nonlinear soft bit K-best detector with variant configurations, we thus defined that the verified detector in MATLAB was the golden model of our design. Figure 3 proves and compares the BER performance of 16-QAM and 64-QAM demodulation with different K values, and Figure 4 shows the BER performance comparison of QPSK and 16-QAM demodulation with the same K values in the case of QRD and SQRD.

Looking at Figure 3 and Figure 4, we can see that when the SNR was between 15 and 35 dB, the BER of the larger K value was 1–2 dB higher than that of the low K value. When the SNR of 16QAM demodulation was higher than 20 dB, the influence of different K values on the BER became smaller. Therefore, in the proposed algorithm, the BER performance will gradually improve with the increase in the K value in the case of a low SNR, while in the case of a high SNR, the influence of different K values will be lessened, and the BER performance will be relatively stable.

**Figure 5:**
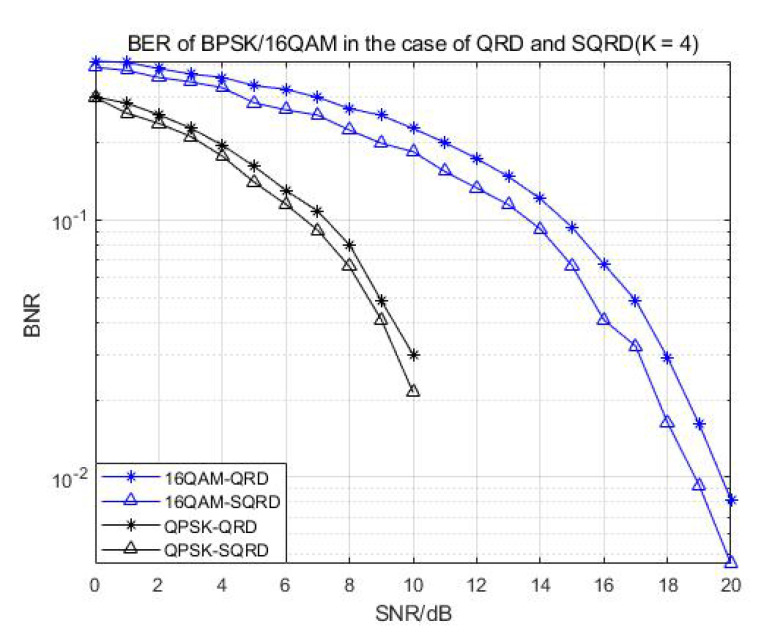
BER of BPSK/16-QAM in the case of QRD and SQRD (K = 4).

**Figure 6:**
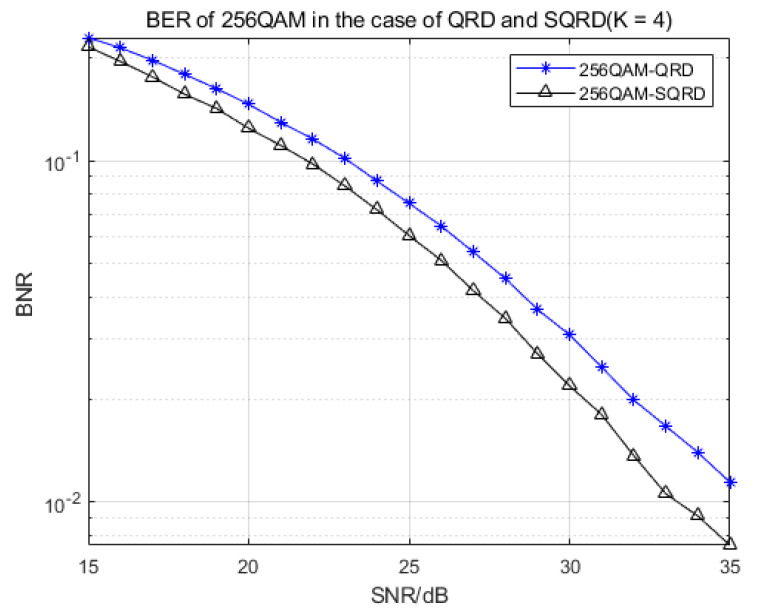
BER of 256-QAM demodulation in the case of QRD and SQRD (K = 4).

Looking at Figure 5 and Figure 6, we can see that under the condition of the same K value, the QRD algorithm and the SQRD algorithm based on the H-column permutation of the channel matrix were simulated and compared. Because the SQRD algorithm minimized the impact of error propagation on subsequent detection, the overall performance of the algorithm was about 1–1.5 dB of gain compared with the traditional QR decomposition algorithm. The results show that the correctness and performances are sufficient for further hardware implementation.

Further BER comparison between the soft and hard bits showed that the soft bit gain of high-order modulation with good channel conditions was 2–3 dB, so both the soft output and hard output were realized in this paper.

The authors of [17] used the K-best algorithm based on SQRD, which is similar to that in this paper, under the same SNR, and the overall performance of the algorithm was about 1.5–2 dB of gain compared with that in [17]. In addition, compared with other published reference algorithm designs, the performance in this paper was very similar to that of the ML detection algorithm and met the requirements of the system.

## 4. Hardware Architecture and Implementation

### 4.1. Algorithm Implementation

The architecture simulator designed in this paper ensured that the low-cost architecture design was consistent with golden model compliance after optimization. The modules in this paper were the sample selector, PED calculator, sorter and LLR calculator. In the selector, the candidate node was selected by configuring the modulation mode and the addressing function. In the PED calculator and sorter, the output was adjusted according to the amount of data to be processed. The LLR calculator was only used to calculate the soft bit information value.

#### 4.1.1. Sample Selector

The function of the module is divided into two parts: first, the data region of the candidate node is divided according to the modulation mode M and the received signal y, and then the candidate nodes are chosen in the selected area in the full constellation table S. When the modulation mode was QPSK or 16QAM, all the constellation points in the full constellation table S were directly selected without going through the data region division, and when the modulation mode was 64QAM or 256QAM, the data region division was needed first, and then point selection was carried out. It should be noted that in this module, the region division of the candidate nodes is based on the estimated received signal $H^{\prime }$ obtained by the preprocessing of MMSE equalization. Table 1 shows the number of nodes that need to be searched by the ML, K-best and proposed algorithm under different modulation modes (with a subcarrier in the case of demodulation with a receiving antenna of four as an example). It can be seen that after the square region division of the candidate nodes, the number of nodes that 64QAM and 256QAM needed to search and calculate for the K-best algorithm was greatly reduced, which saved computing time and hardware overhead.

**Figure 7:**
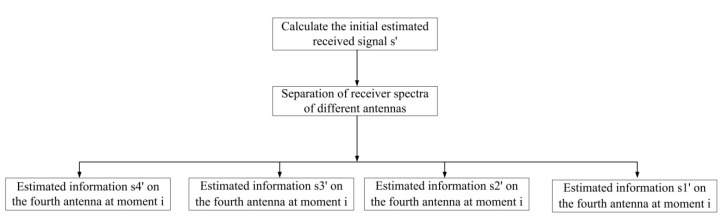
MMSE equalization preprocessing process before selecting points in sample selector.

**Figure 8:**
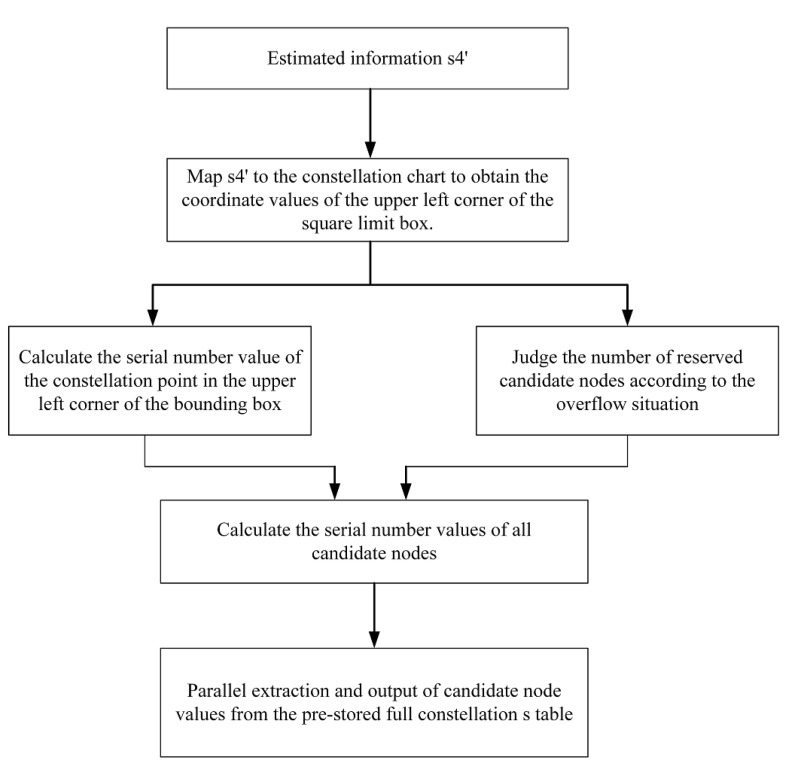
Flowchart of candidate node selection (taking layer 4 as an example).

The flow chart of the module is shown in Figure 7 and Figure 8, and the pseudocode of the algorithm of the sample selector is as follows (Algorithm 1).
**Algorithm 1** Sample selector**Input: **M: Modulation scale; s: Initial estimate s**Output:** Candidate node: Set of each layer candidate node  1: **if** M = 4(QPSK) or 16(16QAM) **then**  2:  Base address = 0;  3:  Select all constellation points as Candidate node;  4: **else**  5:  Calculate Base address based on s value;  6:  Add the corresponding full constellation S table according to M;  7:  Find the candidate node value of the corresponding sequence number in the S table    and store it in candidate node;  8: **end if**  9: **return** candidate node

#### 4.1.2. PED Calculator

The PED value of each layer was calculated according to Equation (7). In order to achieve the accuracy sorting of the simplest data in the minimum time, after the PED value of each candidate node was calculated, it was necessary to carry out large value saturation processing. We needed to find the smallest K PED values (the K value is usually small), and there were many candidate PED values for each layer, so we directly set the upper limit of the PED value, quantified the value within the upper limit (8-bit) and then discarded the larger PED values. This can not only reduce the number of samples but also reduce the overhead of sorting. The number of samples after the large value saturation treatment was 10% lower, and the performance was basically unchanged.

#### 4.1.3. Sorter

In the K-best detection algorithm, a very important step is to sort the calculated PED values. In the proposed algorithm in this paper, the binary data with limited precision and small samples need to be sorted. Most of the traditional sorting methods are bubble sorting and comparison sorting, suffering from large resource overheads and large computing time costs. Therefore, this paper designed a small sample limited precision binary data bit sorting method to complete the sorting function.

**Figure 9:**
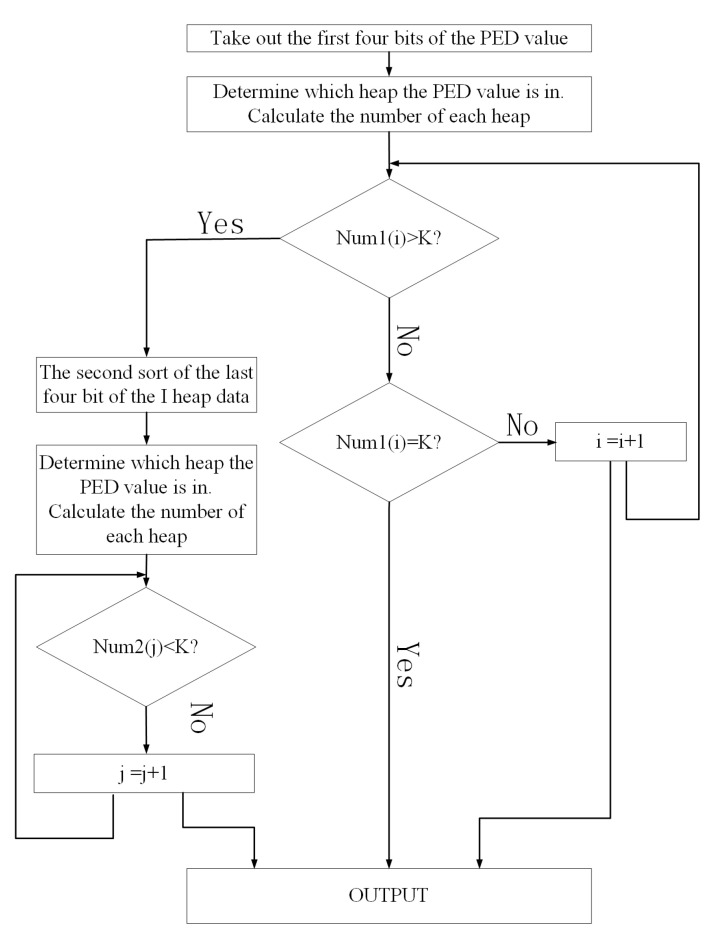
Flowchart of multiple 8-bit data sorting.

To minimize the sorting time, the 8-bit sorting method in this paper used the method of parallel filtering to process the data in clusters at the same time, saving the needed data from small to large. This method only processed the data that met the conditions and discarded the data that did not meet the conditions by constantly updating the conditions, which could reduce the number of times the data was processed. Shown in Figure 9 is a sorter flow chart of a plurality of 8-bit data.

Table 2 compares the common bubble sorting method with the bit sorting method presented in this paper. It can be seen that although the bubble sorting method and the bit sorting method in this paper had considerable stability, the average time required by the bubble sorting method increased exponentially with the increase in the number of samples, while the average time of the bit sorting method used in this paper was stable, and the latter had obvious advantages when the number of samples was large. In addition, in the hardware implementation, the bubble sorting method used subtractors many times when comparing adjacent data, and the bit sorting rule of this paper used multiple AND gates in parallel instead of subtractors to complete the comparison function, which not only reduced the time delay but also further reduced much of the hardware overhead.

The pseudocode of the algorithm of the sorter module is as follows (Algorithm 2).
**Algorithm 2** Sorter**Input**: PED: PED sample values that need to be sorted.  Path: Candidate path corresponding to the sample.  K: The number of samples to be retained after sorting.**Output:** remPED: Reserved PED value.  remPath: Corresponding candidate path. 1: Function First sorting(PED, Path) 2: PED is divided into 2^*n*^ heaps according to the characteristics of pre-$\frac{n}{2}$ bits, and the  number of each heap num1(i) is calculated. 3: **for** i = 1 to 2^*n*^
**do** 4:  **if** num1(i) > K **then** 5:   The num1 data(as PED2) and the paths(as Path2) are sorted for the second time; 6:  **else if** num1(i) < K **then** 7:   The num1 data and the paths as output, i = i + 1; 8:  **else** 9:   The num1 data and the paths as output; 10:  **end if** 11:  NEXT i 12: **end for** 13: Function second sorting(PED2, Path2) 14: PED2 is divided into 2^*n*^ heaps according to the characteristics of rest-$\frac{n}{2}$ bits, and the   number of each heap num2(i) is calculated. 15: **for** i = 1 to 2^*n*^
**do** 16:  **if** num2(i) < K **then** 17:   The num2 data and the paths as output, i = i + 1; 18:  **else** 19:  The num2 data and the paths as output; 20:  **end if** 21:  NEXT i 22: **end for** 23: **return**

### 4.2. Hardware Architecture Design

**Figure 10:**
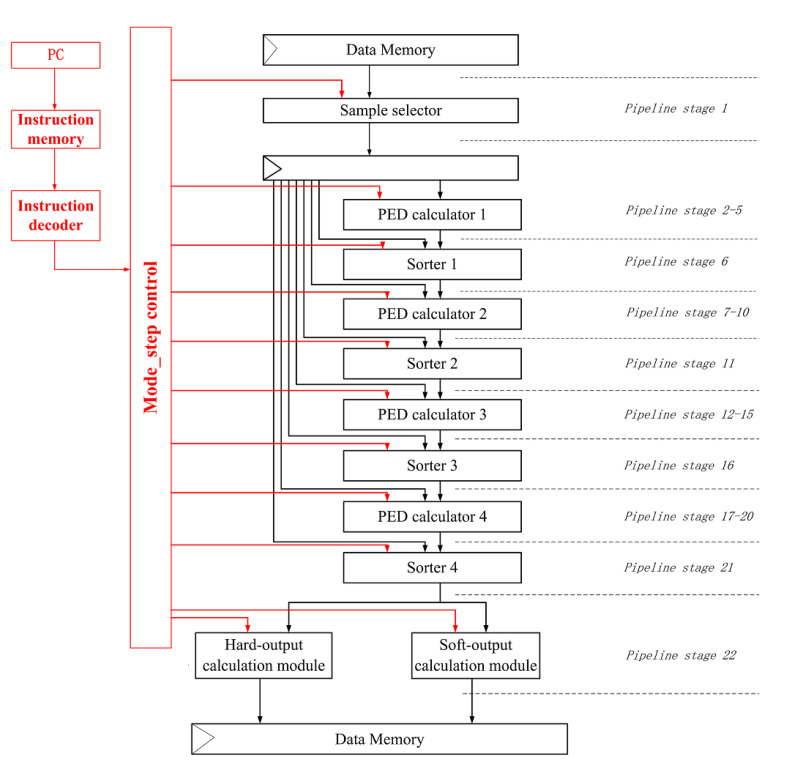
Top-level architecture pipeline chart.

The hardware architecture includes a control path and data path, which are integrated into the same processor module [26]. Starting from the data path, the data from the register are sent to the computing cell array through the distribution logic, the output data of the computing cell array are stored in the memory or buffer, and the reconfigurable parameter memory sends the reconfigurable parameters into the computing cell array. Starting with the control path, the control module inputs the activation signal into the calculation function unit, activates the corresponding calculation module and outputs the activation signal of the next module to the control module, which is transmitted by the control module and activates the next calculation module. The proposed hardware top-level architecture of detector processors is shown in Figure 10.

According to the different functional steps in the algorithm, the calculation function module is divided into several smaller modules. The pipeline structure is implemented by a multi-stage processing unit, and each level performs the function of a module [26]. After calculation, the data as input are forwarded to the next level’s unit, and the activation signal of the next module is transmitted to the control module at the same time. The application of this pipeline can improve data throughput and hardware utilization. The K-best detection algorithm has no backtracking operation and is suitable for pipelining.

### 4.3. Hardware Implementation Results

In the hardware system verification, we performed the following two actions of work. On the one hand, we used the C model to simulate each sub-module at the hardware level and then write the RTL code, compile it in QuartusII, check the correctness of the RTL and finally write the test files, add incentives and test the standard output results so as to verify the correctness of the function. On the other hand, under the condition of ensuring the performance of the algorithm, we used the SMIC 28-nm digital CMOS technology to carry out the hardware comprehensive compilation and layout. After the hardware synthesis and layout, we found the area cost, circuit speed and power consumption report, which were used for comparison.

#### 4.3.1. IC Hardware Layout

**Figure 11:**
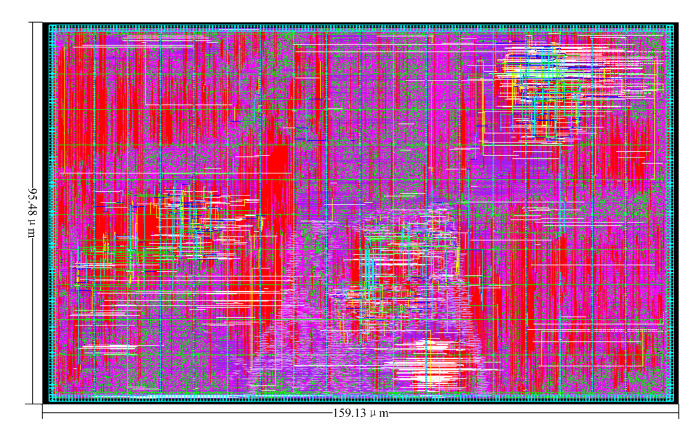
IC hardware layout.

The IC hardware layout is shown in Figure 11.

#### 4.3.2. Hardware Overhead and Comparison

The previously presented K-best MIMO detector was fabricated in SMIC 28-nm technology. The area of the chip was 159.13 μm × 95.48 μm. At the normal 1.2-V core power supply, the detector could work at an 800-MHz clock rate to support a 6400-Mb/s throughput. Table 3 shows the comparison of this paper’s results with other published K-best detectors. The detection throughput in Table 3 is given by [26]

(9) $$\begin{array}{cc} \;\mathrm{Throughput}\; & =f_{c}\times \frac{\log_{2}M\times N_{t}}{T} \end{array}$$

where M is the real constellation size, $N_{t}$ is the antenna size, $f_{c}$ is the clock frequency and *T* is the number of clocks required for each set of data output.

Specifically, the normalized silicon overhead was used as a parameter to compare the capabilities of different detector systems, and this parameter can eliminate the differences between different designs as a unified parameter of measurement. The specific calculation formula is given in Table 3.

Through comparison, we can see that the K-best soft-output signal detector based on SQRD designed in this paper is superior to other designs in the following aspects:

(1) In 64QAM and higher-order modulation, the target algorithm reduces the number of search nodes to 16, which significantly reduces the computing overhead, area and power consumption. At the same time, the MMSE algorithm is used to estimate the initial nodes, which ensures a certain demodulation efficiency while reducing the search space.

(2) The pipeline design of the detector and the prediction of candidate nodes greatly improve the demodulation efficiency. Therefore, the throughput of decoding can be significantly improved.

(3) In the hardware implementation, the design of the detector adopts parallel processing and a pipeline structure at the RTL level so as to achieve the purpose of reducing power consumption. At the same time, the design of the bit sorting method for binary data with limited precision for small samples reduces the use of comparators and reduces the cost of the logic hardware.

In fact, base station chip industries need better designs to achieve both low costs and high performance. However, in the existing designs, some pay more attention to the improvement of performance, which leads to excessive hardware overhead. Some prefer a low hardware overhead, inducing low data throughput. Through the comparison at the end of the article, we can see that the design in this paper has great advantages when using the normalized silicon cost as the comparison standard, which also means that we found better results, which can be used for all base station IC companies. In addition, the design of the detector can be used in the modulation systems of BPSK, QPSK, 16QAM, 64QAM and 256QAM. The detector can choose the demodulation mode and output mode (hard or soft bits) according to the set parameters.

## 5. Conclusions

In this paper, an SQRD-based K-best MIMO detector for 5G micro base stations is proposed which can be configured to complete the detection of high-order modulation (256QAM and 64QAM) and be backward compatible with low-order modulation (16QAM, QPSK, and BPSK) signal detection. At the algorithm level, the MMSE preprocessing method is used to set a 4 × 4 square range limit for the candidate nodes to achieve lower computational complexity, while the performance loss is negligible. At the hardware level, two improvement methods are used: reducing the computing cost by saturating and truncating large values during PED computing and reducing the sorting cost by using the binary bit sorting method for a reduced sample set with finite accuracy. The pipelined structure we proposed can output one subcarrier’s soft information in each clock cycle. In the case of 256QAM modulation, a maximum of 8 soft bits of information can be achieved in a cycle (here, the resolution of the soft bits was 8-bit), which further improves the detection efficiency. Compared with the existing detectors, the area is reduced to 153 K, and the power consumption is only 136 mW while offering a 6400-Mbps throughput when the clock cycle is 800 MHz. This result also shows that under the premise of ensuring near-ideal performance, this design has great advantages over those in the recent literature, which verifies the feasibility of the proposed algorithm and structure. 

## Figures and Tables

**Table 1:** Number of search nodes under different modulation modes (K = n).

Mode	ML	K-Best	K-Best in This Work
QPSK	$4+4^{2}+4^{3}+4^{4}$	4×(3n+1)	4×(3n+1)
16QAM	$16+16^{2}+16^{3}+16^{4}$	16×(3n+1)	16×(3n+1)
64QAM	$64+64^{2}+64^{3}+64^{4}$	64×(3n+1)	16×(3n+1)
256QAM	$256+256^{2}+256^{3}+256^{4}$	256×(3n+1)	16×(3n+1)

**Table 2:** Comparison of bubble sort and bit sort.

Mode	Average Time Complexity	Space Complexity	Stability
Bubble sort	$o(n^{2})$	o(1)	Stable
Bit sort in this paper	o(1)	o(n)	Stable

**Table 3:** Comparison of this paper’s results with other published K-best detectors.

Reference	[12]	[15]	[27]	[28]	[29]	This Work
Modulation Mode	64QAM	64QAM	64QAM	64QAM	64QAM	BPSK∼256QAM
Technology	65 nm	65 nm	65 nm	65 nm	40 nm	SMIC 28 nm
Antennas	4×4	16×16	16×16	4×4	8×8	4×4
Pre-progress	SQR	SQR	SQR	SQR	QR	SQR
Performance	Near-ML	Near-ML	Near-ML	Near-ML	Near-ML	Near-ML
Max Freq. (MHz)	450	588	625	500	641	800
Throughput (Mbps)	300	3528	3910	368	3846	6400
Area	184 K	3720 K	5891 K	1055 K	3873 K	153 K
Power (mW)	138.2	1831	548	307	1555	136
NAE	1.63	15.9	10.56	0.34	2.44	18.01

Normalized area efficiency (NAE) [13,26]: $\mathrm{NAE}=\frac{\mathrm{Throughput}}{\mathrm{Area}}\times \frac{\mathrm{Tech}}{S}\times \frac{N^{2}}{P}$. All designs are scaled to 65 nm, where S = 65 nm. All designs are scaled to 4 × 4: $\frac{N^{2}}{P}=\frac{N^{2}}{4\times 4}$ .

## Data Availability

Not applicable.
